# Short-term effects of obesity surgery versus low-energy diet on body composition and tissue-specific glucose uptake: a randomised clinical study using whole-body integrated ^18^F-FDG-PET/MRI

**DOI:** 10.1007/s00125-024-06150-3

**Published:** 2024-04-24

**Authors:** Jan W. Eriksson, Maria J. Pereira, Christakis Kagios, Sofia Kvernby, Elin Lundström, Giovanni Fanni, Martin H. Lundqvist, Björn C. L. Carlsson, Magnus Sundbom, Sambit Tarai, Mark Lubberink, Joel Kullberg, Ulf Risérus, Håkan Ahlström

**Affiliations:** 1 Department of Medical Sciences, Clinical Diabetology and Metabolism, Uppsala University, Uppsala, Sweden; 2 Department of Surgical Sciences, Molecular Imaging and Medical Physics, Uppsala University, Uppsala, Sweden; 3 Department of Surgical Sciences, Radiology, Uppsala University, Uppsala, Sweden; 4 Research and Early Development, Cardiovascular, Renal and Metabolism, BioPharmaceuticals R&D, AstraZeneca, Gothenburg, Sweden; 5 Department of Surgical Sciences, Surgery, Uppsala University, Uppsala, Sweden; 6 Antaros Medical, Mölndal, Sweden; 7 Department of Public Health and Caring Sciences, Clinical Nutrition and Metabolism, Uppsala University, Uppsala, Sweden

**Keywords:** Insulin resistance, Low-energy diet, Obesity surgery, Tissue-specific glucose turnover, Weight loss, Whole-body PET-MRI

## Abstract

**Aims/hypothesis:**

Obesity surgery (OS) and diet-induced weight loss rapidly improve insulin resistance. We aim to investigate the impact of either Roux-en-Y gastric bypass (RYGB) or sleeve gastrectomy (SG) surgery compared with a diet low in energy (low-calorie diet; LCD) on body composition, glucose control and insulin sensitivity, assessed both at the global and tissue-specific level in individuals with obesity but not diabetes.

**Methods:**

In this parallel group randomised controlled trial, patients on a waiting list for OS were randomised (no blinding, sealed envelopes) to either undergo surgery directly or undergo an LCD before surgery. At baseline and 4 weeks after surgery (*n*=15, 11 RYGB and 4 SG) or 4 weeks after the start of LCD (*n*=9), investigations were carried out, including an OGTT and hyperinsulinaemic–euglycaemic clamps during which concomitant simultaneous whole-body [^18^F]fluorodeoxyglucose-positron emission tomography (PET)/MRI was performed. The primary outcome was HOMA-IR change.

**Results:**

One month after bariatric surgery and initiation of LCD, both treatments induced similar reductions in body weight (mean ± SD: −7.7±1.4 kg and −7.4±2.2 kg, respectively), adipose tissue volume (7%) and liver fat content (2% units). HOMA-IR, a main endpoint, was significantly reduced following OS (−26.3% [95% CI −49.5, −3.0], *p*=0.009) and non-significantly following LCD (−20.9% [95% CI −58.2, 16.5). For both groups, there were similar reductions in triglycerides and LDL-cholesterol. Fasting plasma glucose and insulin were also significantly reduced only following OS. There was an increase in glucose AUC in response to an OGTT in the OS group (by 20%) but not in the LCD group. During hyperinsulinaemia, only the OS group showed a significantly increased PET-derived glucose uptake rate in skeletal muscle but a reduced uptake in the heart and abdominal adipose tissue. Both liver and brain glucose uptake rates were unchanged after surgery or LCD. Whole-body glucose disposal and endogenous glucose production were not significantly affected.

**Conclusions/interpretation:**

The short-term metabolic effects seen 4 weeks after OS are not explained by loss of body fat alone. Thus OS, but not LCD, led to reductions in fasting plasma glucose and insulin resistance as well as to distinct changes in insulin-stimulated glucose fluxes to different tissues. Such effects may contribute to the prevention or reversal of type 2 diabetes following OS. Moreover, the full effects on whole-body insulin resistance and plasma glucose require a longer time than 4 weeks.

**Trial registration:**

ClinicalTrials.gov NCT02988011

**Funding:**

This work was supported by AstraZeneca R&D, the Swedish Diabetes Foundation, the European Union’s Horizon Europe Research project PAS GRAS, the European Commission via the Marie Sklodowska Curie Innovative Training Network TREATMENT, EXODIAB, the Family Ernfors Foundation, the P.O. Zetterling Foundation, Novo Nordisk Foundation, the Agnes and Mac Rudberg Foundation and the Uppsala University Hospital ALF grants

**Graphical Abstract:**

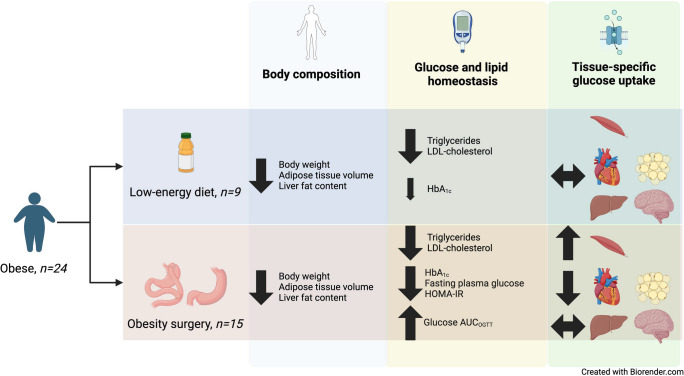

**Supplementary Information:**

The online version of this article (10.1007/s00125-024-06150-3) contains peer-reviewed but unedited supplementary material.


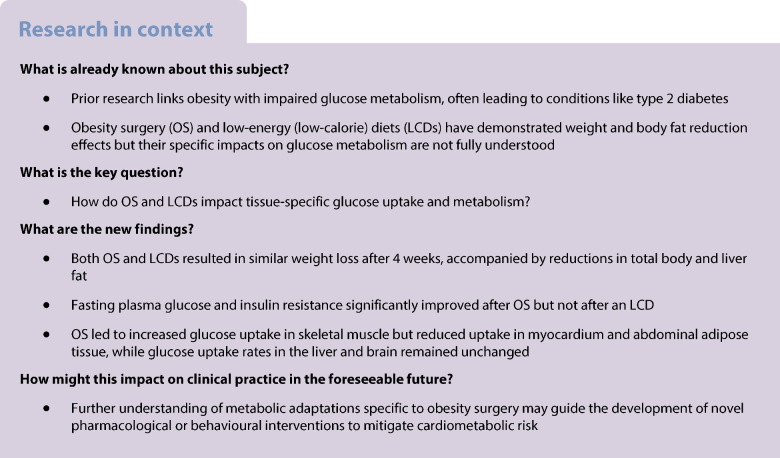


## Introduction

Over recent decades, the prevalence of obesity has increased dramatically, reaching pandemic levels worldwide, there being over 1.9 billion adults with overweight or obesity in 2016 [1]. Overweight and obesity are characterised by an excessive accumulation of body fat, which, together with an unfavourable distribution, significantly contributes to various health issues, including perturbed glucose and lipid metabolism in insulin-sensitive tissues, leading to type 2 diabetes and dyslipidaemia. Additionally, it increases the risk for several comorbidities, including hypertension, major cardiovascular events, renal impairment and several cancer forms, as well as hepatobiliary, musculoskeletal and psychiatric diseases [1–4]. Effective interventions are crucial to combat obesity and its associated comorbidities. Pharmaceutical options such as semaglutide and tirzepatide, which mimic gastrointestinal (GI) hormones, show promise, although their long-term efficacy and safety warrant further investigation [5]. Obesity surgery (OS) and diets low in energy (low-calorie diets; LCDs) have demonstrated substantial weight loss and reduced obesity-related morbidity and mortality [6, 7].

Several surgical options, including Roux-en-Y gastric bypass (RYGB) and sleeve gastrectomy (SG), involve gastrointestinal tract rearrangements and reduce energy intake and uptake [8]. RYGB is particularly efficient for long-term weight loss [9, 10] and prevention or reversal of type 2 diabetes [11, 12]. OS also mitigates other obesity-related comorbidities, such as CVD, fatty liver disease and sleep apnoea [7–9, 13, 14], and improves quality of life [7, 13]. The cardiometabolic benefits of OS (e.g. a rapid effect on glucose homeostasis) are partly independent of weight loss [14]. Thus, OS is commonly termed metabolic surgery [12, 15], although the mechanisms for its diverse benefits remain incompletely understood [14], with some studies proposing a potential role of the brain [16, 17].

On the other hand, LCDs are commonly recommended as the primary approach for weight loss [18]. Several diet principles aim to reduce daily energy intake [19]. While nutritionally balanced and individually customised LCDs can improve glycaemic control, liver fat content, BP and lipid profiles [20], long-term adherence and weight maintenance are challenging [9, 21–23].

Limited evidence exists of the short-term comparative effectiveness of diet- and surgery-based interventions for weight loss and their effects on glucose and lipid handling in different tissues. Therefore, this RCT aimed to investigate the metabolic effects observed 4 weeks after OS or the initiation of LCD, with a special focus on tissue-specific glucose uptake and metabolism. We combined traditional gold-standard metabolic assessments, such as the OGTT and the hyperinsulinaemic–euglycaemic clamp, with simultaneous [^18^F]fluorodeoxyglucose (^18^F-FDG) positron emission tomography (PET) and MRI to assess glucose turnover in individual tissues and body fat content and distribution. We hypothesised that OS would rapidly improve insulin sensitivity, as evidenced by the glucose clamp test, largely through a reduction in endogenous glucose production (EGP).

## Methods

### Participants

**Fig. 1:**
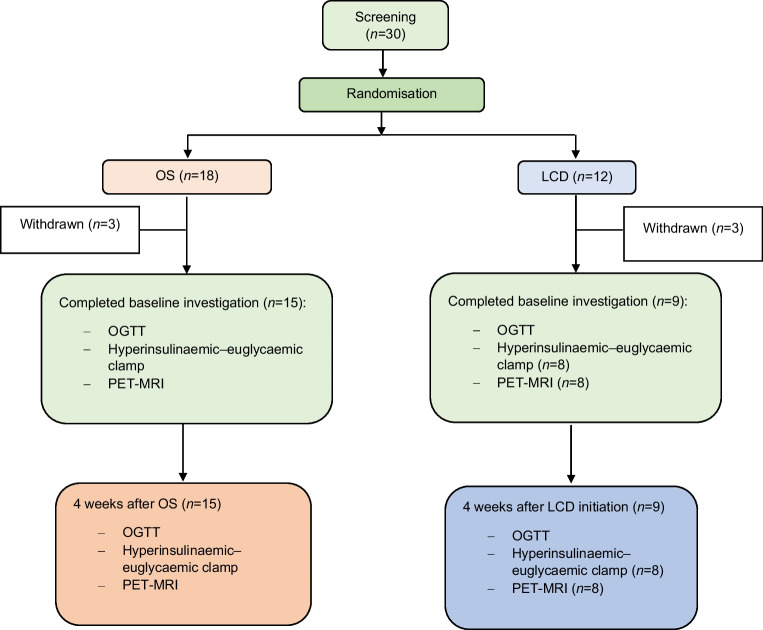
CONSORT diagram. Flow chart showing participant progression. For details, see Methods

Thirty non-diabetic patients (28 women, 2 men (self-reported); BMI 35–45 kg/m^2^) planned to undergo an obesity surgery were recruited at the Department of Endocrinology and Diabetes, Uppsala University Hospital, Uppsala, Sweden, during a pre-surgical endocrinological assessment. Exclusion criteria included diabetes, other endocrine disorders (except well-treated hypothyroidism), cancer, previous cardiovascular events, untreated sleep apnoea, major illnesses and pregnancy. The study consisted of a parallel-group randomised controlled trial. The participants were randomly assigned to two arms: (1) OS (RYGB or SG by patient decision) without presurgical diet change; or (2) LCD for 4 weeks, followed by OS. The OS/LCD allocation ratio was initially 1:1, later altered to 2:1. The allocation sequence was obtained using sequentially numbered, sealed envelopes, which were kept by a research nurse. Neither study participants nor caregivers and study investigators were blind to the group assignment. Six participants withdrew before or during the baseline investigations, due to reasons such as back pain, claustrophobia and lack of venous access (Fig. 1). Ultimately, 15 participants in the OS arm and nine in the LCD arm completed the 4 week intervention and follow-up, albeit one could not complete clamps with PET/MRI (LCD group; due to claustrophobia). The study participants were representative of the population undergoing obesity surgery in terms of sex and age distribution. The study adhered to Consolidated Standards of Reporting Trials (CONSORT) guidelines [24]. The Regional Ethics Review Board in Uppsala approved the study (Dnr 2015/514 with amendment 2015/514/1). All participants provided written informed consent (ClinicalTrials.gov registration no. NCT02988011) and investigations followed the principles of the Declaration of Helsinki (2008).

### Experimental design

Participants were assessed at baseline and 4 weeks post LCD or surgery. In the surgery group, 15 participants were offered RYGB or SG. The LCD consisted of liquid meal (Modifast; Täby, Sweden) replacements for 4 weeks, providing 4600 kJ (1100 kcal) energy intake per day. Both groups aimed for 8–10% weight loss [22, 25]. The primary outcome was HOMA-IR change. Secondary outcomes included anthropometric measures (weight, waist and hip circumference, and body fat % measured by bioimpedance using Tanita BC-418 [Tanita Corporation, Tokyo, Japan]), blood tests, subcutaneous adipose tissue (SAT) needle biopsy, 2 h 75 g OGTT, hyperinsulinaemic–euglycaemic clamp with glucose uptake assessment with ^18^F-FDG-PET, and 24 h physical activity and energy expenditure. See electronic supplementary material (ESM) Methods for additional details.

### Hyperinsulinaemic–euglycaemic clamp

A hyperinsulinaemic–euglycaemic clamp was performed, as previously reported to be compatible with whole-body PET/MRI [26, 27]. In brief, human insulin (Actrapid; Novo Nordisk, Copenhagen, Denmark) was infused at 56 mU (m^2^ body surface)^−1^ min^−1^, and plasma insulin levels were raised to about 700 pmol/l. A variable glucose infusion (200 mg/ml) was simultaneously adjusted to maintain stable plasma glucose at 5.6 mmol/l. The *M* value, reflecting whole-body insulin sensitivity, was derived from the glucose infusion rate during the clamp steady state (60–120 min) and calculated as glucose infusion rate per lean body mass (LBM) (mg [kg LBM]^−1^ min^−1^).

### PET/MRI acquisition

We used a whole-body integrated simultaneous 3.0 T PET/MRI system (Signa PET/MR; GE Healthcare, Waukesha, WI, USA) following an established protocol [27, 28]. Initially, 4 MBq of ^18^F-FDG per kg of body weight was injected for a 10 min dynamic PET scan focusing on early ^18^F-FDG dynamics in the thorax. Subsequently, five static whole-body PET scans, covering head to toe, were conducted with ten bed positions, each lasting 30 s. Simultaneously acquired water-fat (Dixon) MRI data, using vendor-specific magnetic resonance attenuation correction (MRAC) sequence, facilitated PET data attenuation correction and whole-body tissue segmentation. MRAC generated two distinct whole-body image datasets: a water signal image; and a fat signal image. Comprehensive corrections for quantitative PET evaluation, including random and scatter corrections, were executed. After PET/MRI scans, we performed two dedicated water-fat MRI acquisitions of the liver and pancreas using a 3D six-echo gradient-echo acquisition and a vendor-specific water-fat reconstruction method (iterative decomposition of fat and water with echo asymmetry and least squares estimation [IDEAL-IQ) [29]). This sequence produced quantitative fat fraction images with voxel intensities representing the fraction of the fat signal to the total water and fat signal.

### PET/MRI-derived quantifications

Quantitative ^18^F-FDG net glucose influx rate Ki images were generated. Whole-body glucose disposal (Rd), EGP and specific tissue glucose metabolic rate (MRglu) were calculated based on ^18^F-FDG kinetics, tissue lumped constants and adjustment for urinary and blood ^18^F-FDG, as previously reported [26, 29]. Adipose and non-adipose tissue volumes were quantified from whole-body water-fat MRI using automated image analysis. Liver and pancreatic fat % were quantified from manually delineated fat fraction maps. See ESM Methods for details.

### Statistical analyses

Data are displayed as mean ± SD unless otherwise indicated. All data were first checked for normality using Shapiro–Wilk test and normal distribution of the residuals was analysed by visual validation of Q–Q plots. Homoscedasticity was deemed acceptable after visual inspection of residuals vs predicted values. Non-normally distributed data were log-transformed before analysis. Back-transformation to the original scale was made and results display geometric mean and CI (95% CI). Group differences at baseline between the surgery and LCD group and changes from baseline within a group were analysed using independent *t* test and paired *t* test, respectively. Analyses of change from baseline between the groups (surgery vs LCD) were assessed with ANCOVA adjusted for the outcome baseline value. Comparison between the number of participants with normoglycaemia or prediabetes between OS and LCD was performed with Fisher’s Exact test. For this exploratory research focusing on potential mechanisms there was no formal prespecified power requirement. However, power analyses based on previous studies [30, 31], indicate that the current sample size in the LCD group (*n*=9) gives 80% power to detect a change from baseline in fasting plasma glucose of 0.53 mmol/l and HOMA-IR of 2.2 units, with α=0.05. The larger OS group (*n*=15) provides more than 80% power for such changes. For the comparison of LCD vs OS groups, there is an 80% estimated power to detect a difference in effects on FPG of 0.6 mmol/l and on HOMA-IR of 2.6 units, respectively. A *p* value <0.05 was considered statistically significant, with no multiple testing correction. All data were analysed with IBM SPSS software version 23 (USA) and GraphPad Prism 10.0.2 (USA).

## Results

In total, 24 participants completed the study: 15 were randomised to OS (one male, 14 female) and nine to LCD (one male, eight female) (Fig. 1). Of the 15 participants in the OS group, 11 underwent RYGB and 4 SG. Anthropometric and other clinical characteristics of the cohort at baseline and after intervention are shown in Table 1. The two groups were very similar in age, sex distribution, BMI, waist-to-height ratio and body fat %. In addition, they presented comparable glucose metabolism characteristics, including HbA_1c_, fasting glucose and *M* value at baseline. However, the LCD group had higher levels of fasting triglycerides and lower aspartate aminotransferase (AST) levels and hip circumference (Table 1). There were no serious or unexpected adverse effects following either of the interventions, and all participants completed the study. During post-surgery OGTT, nine participants experienced early dumping symptoms, e.g. nausea, blood pressure drop and diarrhoea, and this is an expected and well-known phenomenon after RYGB.

**Table 1:** Clinical and biochemical characteristics before and after OS or LCD

Characteristic	OS (*n*=15)		LCD (*n*=9)		*p* value (surgery vs LCD)^a^
	Pre	Post	Pre	Post	
Anthropometrics					
Sex, male/female	1/14	-	1/8	-	
Age, years	43±11	-	41±8	-	
BMI, kg/m^2^	40.7±2.5	37.6±2.3***	38.4±2.8	35.5±2.7***	0.917
Weight, kg	110.6 (106.8, 114.7)	102.1 (98.8, 105.5)***	108.4 (97.6, 120.2)	100.3 (90.0, 111.7)***	0.658
Body weight loss, %	-	7.7±1.4	-	7.4±2.2	0.701
Waist circumference, cm	117.4±6.5	111.3±8.4***	119.8±9.3	113.2±8.6**	0.795
Hip circumference, cm	129.2±8.6	125.2±7.2**	121.0±9.1^†^	116.2±6.0*	0.057
Waist/hip ratio	0.91±0.09	0.89±0.10	0.99±0.11	0.98±0.09	0.723
LBM, %^b^	50.5 (48.8, 52.3)	51.9 (49.9, 54.1)*	54.7 (50.0, 59.8)	56.0 (51.2, 61.3)	0.958
Body fat, %^b^	49.3 (47.3, 51.3)	47.8 (45.5, 50.2)*	44.5 (39.6, 50.1)	43.1 (37.8, 49.2)	0.958
Heart rate, beats/min	65±12	65±9	74±8	68±11	0.471
Systolic BP, mmHg	132±15	122±11**	140±13	128±15***	0.746
Diastolic BP, mmHg	74±12	72±10	82±16	77±12	0.406
Glucose metabolism					
HbA_1c_, mmol/mol	35.3±3.4	31.1±2.4***	35.4±4.8	33.6±3.6*	0.007
HbA_1c_, %	5.38±0.31	5.00±0.22***	5.39±0.44	5.22±0.33*	0.006
Fasting plasma glucose, mmol/l	5.6±0.5	5.2±0.4**	6.0±1.0	5.6±0.5	0.771
HOMA-IR	2.4 (1.6, 3.7)	1.6 (1.1, 2.2)**	4.9 (2.7, 9.0)	3.3 (2.1, 5.1)	0.080
Fasting S- insulin, pmol/l	67.9 (44.5, 103.6)	47.0 (33.4, 66.2)*	128.0 (72.9, 224.9)	92.0 (60.3, 140.3)	0.095
Clamp insulin, pmol/l	908±177	743±170**	867±206	857±150	0.047
2 h post-OGTT glucose^c^, mmol/l	7.4±1.5	7.0±1.7	8.2±1.4	8.0±1.6	0.386
Insulinogenic index^c^	1.5±0.7	1.0±0.5	2.1±1.3	1.3±0.8**	0.591
Disposition index^c^	4.2 (3.0, 5.9)	2.9 (1.9, 4.5)*	3.4 (1.6, 7.2)	2.6 (1.5, 4.5)	0.229
Matsuda index^c^	3.2 (2.1, 4.7)	3.3 (2.5, 4.4)	1.9 (1.0, 3.4)	2.7 (1.7, 4.3)	0.574
*M* value, mg (kg LBM)^−1^ min^−1^	8.6±4.3	7.4±2.5	7.9±5.4	6.0±2.5	0.188
Clinical biochemistry					
Plasma creatinine, μmol/l	68±8	63±8*	66±9	71±9*	0.002
Plasma ALT, μKat/l	0.47±0.15	0.51±0.27	0.45±0.30	0.47±0.27	0.739
Plasma AST, μKat/l	0.63±0.29	0.53±0.29*	0.40±0.09^†^	0.40±0.10	0.371
Plasma ALP, μKat/l	1.21±0.29	1.16±0.28	1.14±0.29	1.07±0.24	0.606
Plasma C-reactive protein, mg/l	7.0±4.9	3.7±2.1**	3.7±2.4	2.9±2.7	0.492
Plasma cholesterol, mmol/l	4.6±1.1	3.6±0.7***	5.2±1.0	3.9±0.6***	0.923
Plasma HDL-cholesterol, mmol/l	1.24±0.32	0.99±0.23***	1.16±0.26	0.99±0.15*	0.488
Plasma LDL-cholesterol, mmol/l	2.9±0.8	2.2±0.5***	3.5±0.8	2.5±0.6**	0.966
Plasma triglycerides, mmol/l	1.09 (0.87, 1.37)	0.93 (0.81, 1.07)*	1.60 (1.19, 2.14)^†^	1.16 (1.04, 1.29)*	0.243
Physical activity					
Total physical activity^d^					
Light, min	754±237	666±145	935±248	635±110	0.538
Moderate, min	575±351	586±228	905±261^†^	746±298	0.105
Vigorous, min	26±19	17±17	57±34^†^	45±46	0.164
Energy expenditure, kJ/day (kcal/day)	11,970±2038 (2861±487)	11,184±1686 (2673±403)	14,527±2272 (3472±543)^†^	12,008±2682 (2870±641)	0.167

Data are presented as mean ± SD if normally distributed, otherwise geometric mean and 95% CI

^a^*p* values for change from baseline in surgery vs LCD group, ANCOVA test

^b^Determined by bioimpedance

^c^During OGTT

^d^Sum in min/day spent in different physical activity categories measured by the use of accelerometers during four consecutive days (surgery *n*=15 pre, *n*=10 post; LCD *n*=7 pre, *n*=4 post)

^*^p<0.05, **p<0.01, ***p<0.001 for pre vs post within the group, paired *t* test

^†^*p*<0.05 for pre-surgery vs pre LCD, independent sample *t* test

ALP, alkaline phosphatase; ALT, alanine aminotransferase

### Clinical effects on anthropometric measures

**Fig. 2:**
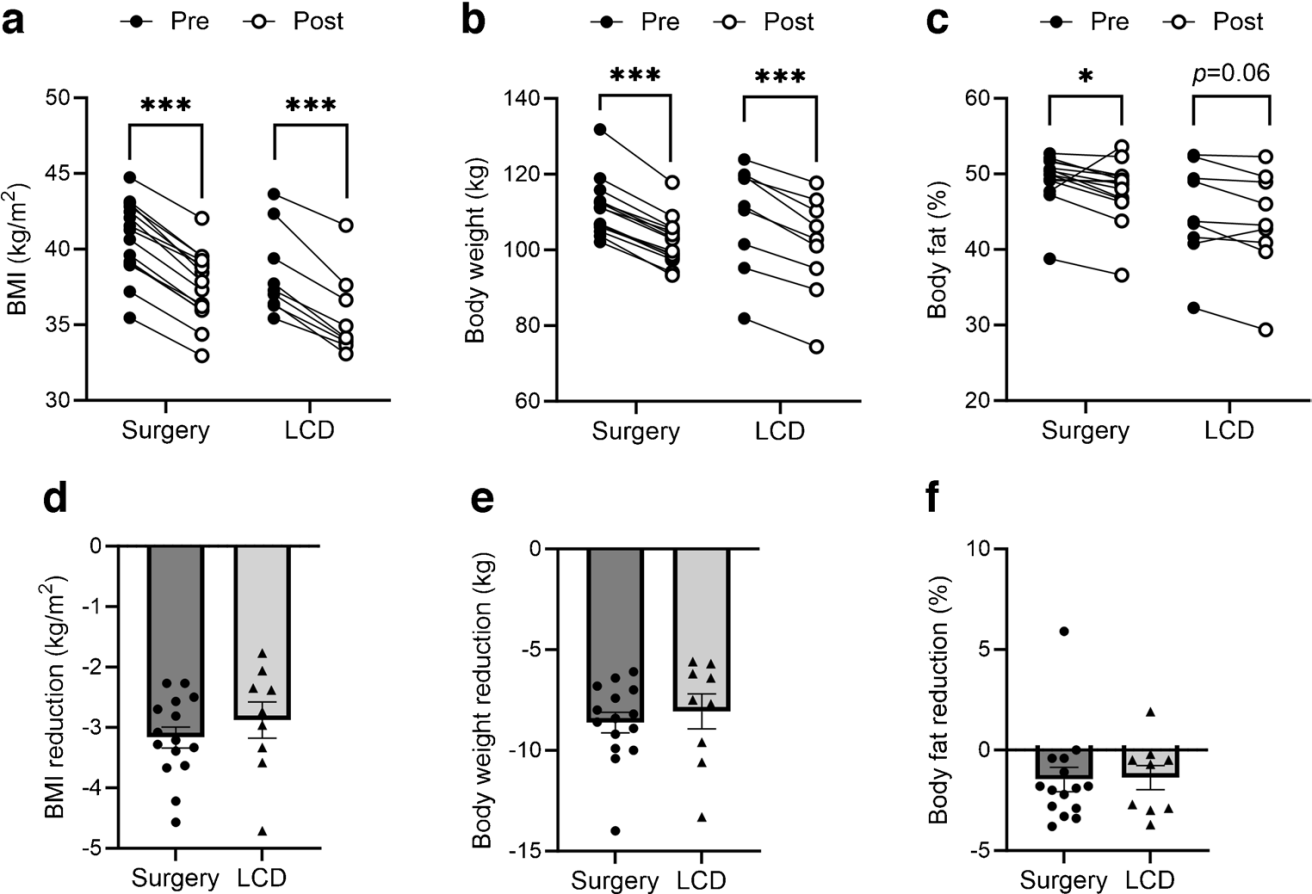
Anthropometric effects of OS (surgery) and LCD on BMI (**a**, **d**), body weight (**b**, **e**) and body fat % (**c**, **f**). Data are means ± SEM; individual values as indicated. Statistical methods and significances are given in Table 1. *p<0.05 and ***p<0.001

Surgery and LCD led to comparable reductions in body weight and BMI (Table 1, Fig. 2). In both groups, the mean weight loss was 8–9 kg (7–8%) over the study period of 4 weeks. Accordingly, waist circumference was decreased by ~6 cm in both groups (Table 1). Moreover, body fat %, as assessed by bioimpedance analyses, was significantly decreased by OS and was decreased (albeit not significantly) by LCD (Table 1, Fig. 2c). There was a corresponding increase in LBM %, which was significant in the OS group (Table 1). Systolic BP was significantly reduced, and diastolic pressure was non-significantly reduced, by OS and LCD to a similar degree.

The level of physical activity assessed by 24 h accelerometer monitoring did not show any significant change after either OS or LCD and, thus, there were no differences between the groups. Further, the estimated 24 h energy expenditure (representing normal life, resting and active) did not show any differences between OS and LCD (Table 1). However, these analyses could be performed only in a subset of participants and therefore must be interpreted with caution.

### Clinical effects on glucose and lipid levels and clinical biochemistry

**Fig. 3:**
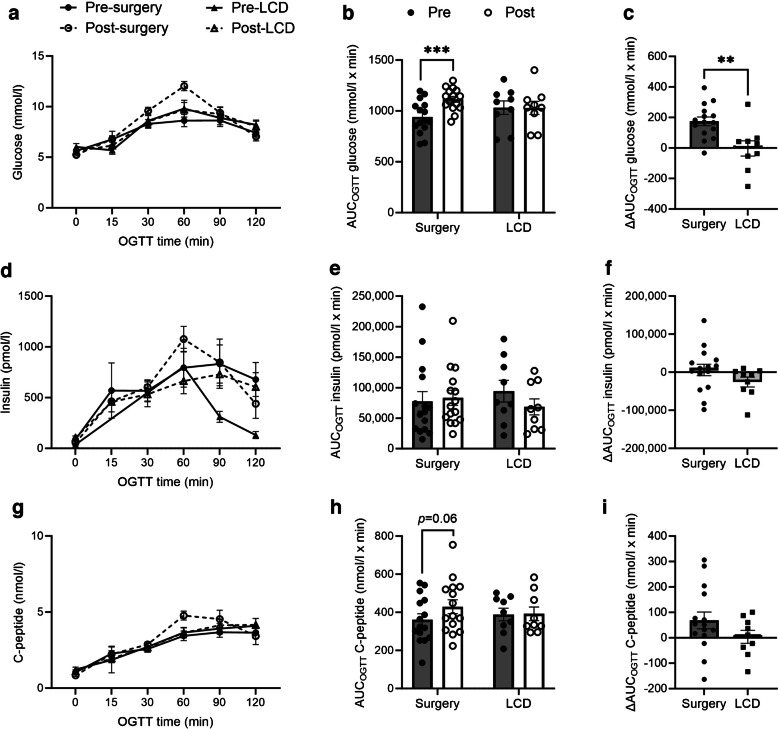
OGTT responses to OS (surgery) and LCD for glucose (**a**–**c**), insulin (**d**–**f**) and C-peptide (**g**–**i**). Data are mean ± SEM; individual values as indicated. Statistical method and significances are given in Table 1. **p<0.01 and ***p<0.001

**Fig. 4:**
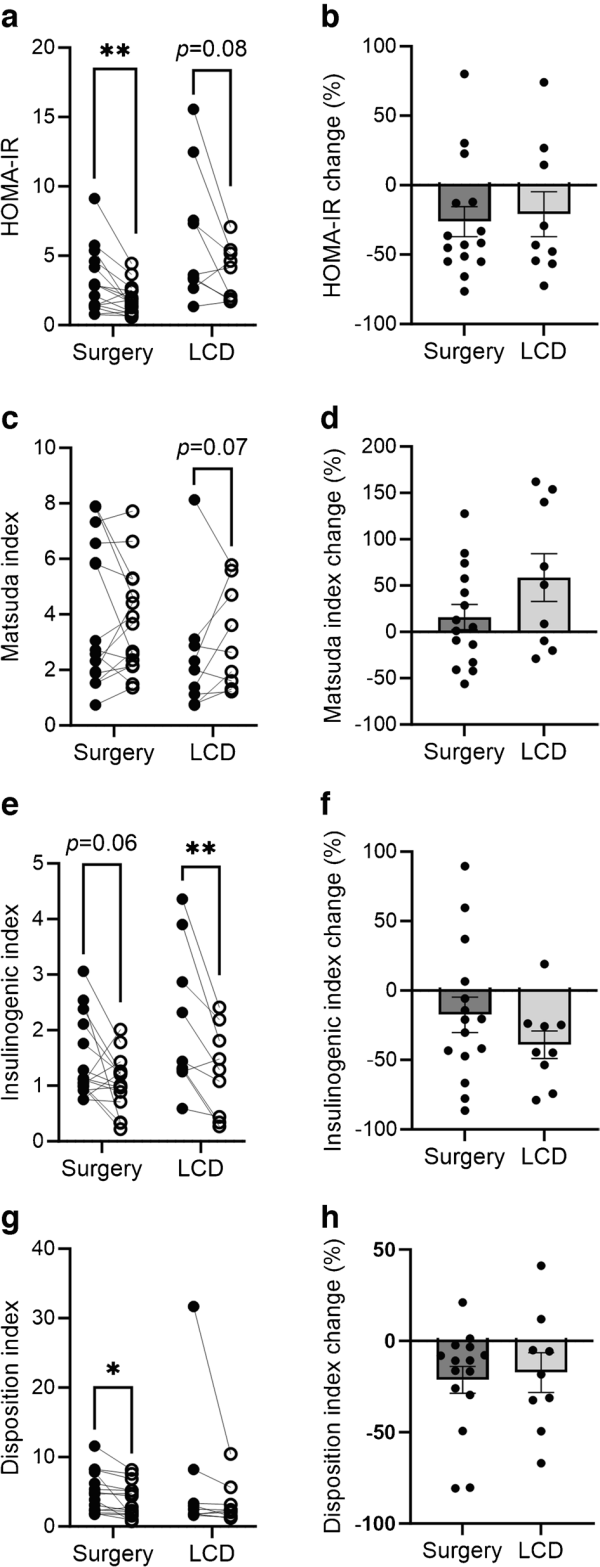
Impact of OS (surgery) and LCD on indices of insulin sensitivity and secretion, in the fasting state (**a**, **b**) or during OGTT (**c**–**h**). Data are mean ± SEM of % change; individual values as indicated. *p<0.05, **p<0.01

Circulating glucose levels, reflected by HbA_1c_, were reduced more markedly by surgery than LCD at 4 weeks (Table 1), with the change from baseline differing significantly when comparing OS with LCD (*p*<0.01). Additionally, fasting glucose and insulin were reduced after OS (*p*<0.01) but not after LCD, with no significant differences in changes from baseline when comparing OS with LCD. The AUC for glucose, insulin and C-peptide concentrations were obtained during an OGTT (AUC_OGTT_; Fig. 3). Glucose AUC_OGTT_ was increased by 20% after OS (*p*<0.001) but not after LCD, and this effect was independent of weight loss (%). Insulinogenic index was slightly reduced after surgery and significantly reduced in the LCD group (Fig. 4e,f), whereas insulin AUC_OGTT_ was unaffected by either of the interventions (Table 1, Fig. 3d–f). The lipid levels, including fasting plasma cholesterol, HDL-cholesterol, LDL-cholesterol and triglycerides were reduced in both groups with no significant difference between the groups.

**Fig. 5:**
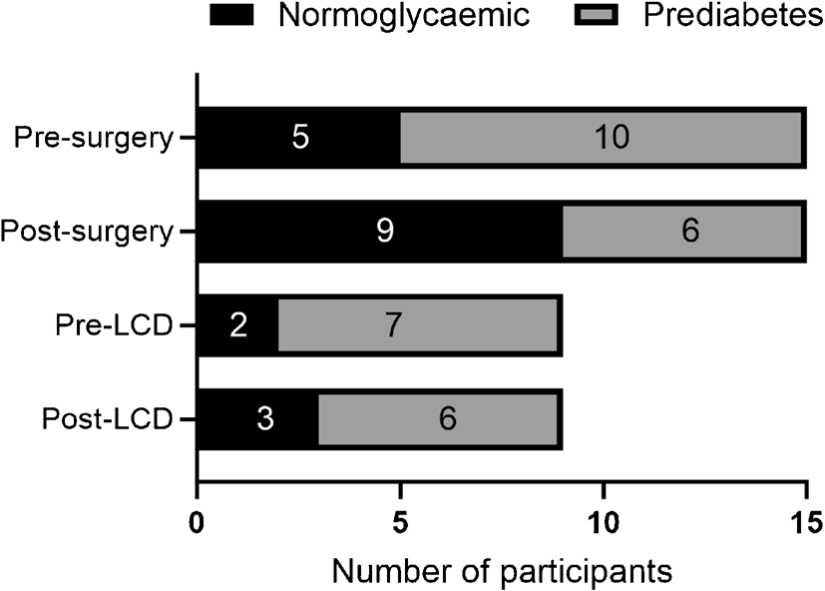
Number of participants with normoglycaemia (black) or prediabetes (impaired fasting glucose or impaired glucose tolerance, grey) before and after OS and LCD

At baseline, seven individuals in the LCD group were diagnosed with prediabetes (either impaired fasting glucose or impaired glucose tolerance) and one of them reversed to a normoglycaemic condition following the diet (Fig. 5). Ten individuals in the OS group had prediabetes before surgery, and four of them reverted to normoglycaemia after the intervention. There was a nominally greater (but not significantly so), proportion of participants with prediabetes that converted to normoglycaemia (40%) in the OS group than in the LCD group (14%).

C-reactive protein and AST were reduced by surgery but not by LCD (Table 1). The impact of OS on reduction of C-reactive protein was independent of weight loss. The interventions had different effects on creatinine (*p*=0.002), with OS reducing levels but LCD increasing levels (*p*<0.05).

### Glucose homeostasis indices

Neither intervention significantly affected whole-body insulin sensitivity at 4 weeks, as assessed by the clamp-derived *M* value (Table 1). HOMA-IR was significantly reduced following OS (−26.3% [95% CI −49.5, −3.0], *p*=0.009); the reduction was not statistically significant following LCD (−20.9% [95% CI −58.2, 16.5]). Similar results were found for disposition index, indicating a possible insulin sensitisation effect. There were no consistent effects on the Matsuda index during a glucose load, and no significant difference between interventions regarding these indices could be detected (Table 1, Fig. 4).

### Body and tissue composition assessed by MRI

Both OS and LCD caused significant decreases in magnetic resonance-derived estimates of whole-body, adipose tissue and non-adipose tissue volumes (all *p*<0.001, Table 2). Adipose tissue volume was comparably decreased by around 7% after both interventions. However, OS led to a greater decrease in non-adipose tissue volume (*p*<0.01), and this was independent of weight loss. Both surgery and LCD markedly decreased liver fat % by approximately 2% units. Neither intervention affected pancreas fat % (Table 2).

**Table 2:** Magnetic resonance-derived body volumes and FDG-PET-derived glucose turnover and uptake rates before and after OS or LCD

Characteristic	OS (*n*=15)		LCD (*n*=9)		*p* value
	Pre	Post	Pre	Post	OS vs LCD^a^
Body volumes, l					
Whole-body volume	103.3±5.9	95.8±5.2***	98.4±13.0	91.8±12.0***	0.369
Adipose tissue volume	67.5±3.5	62.6±3.2***	60.3±9.6	55.3±8.5***	0.116
Non-adipose tissue volume	35.2±3.5	32.7±3.0***	37.7±8.2	36.1±8.0***	0.009
Tissue fat percentage					
Liver	6.1 (4.5, 11.6)	4.0 (3.0, 7.1)***	6.0 (1.8, 15.5)	4.2 (0.63, 12.1)**	0.289
Pancreas	9.3 (5.9, 18.9)	10.1 (6.6, 18.4)	8.0 (4.5, 14.2)	10.3 (7.5, 14.6)	0.251
Whole-body glucose turnover, µmol (kg LBM)^−1^ min^−1b^					
GIR	48.0±24.1	41.1±13.8	43.8±30.2	33.5±14.0	0.183
EGP	12.2±7.6	14.2±8.4	11.4±12.4	13.3±4.7	0.802
Rd	60.2±20.8	55.3±13.1	55.1±23.2	46.8±16.6	0.152
Tissue glucose uptake, µmol (100 ml tissue)^−1^ min^−1^					
MRglu brain	11.2±3.8	11.7±3.9	11.5±4.8	14.2±1.9	0.079
MRglu liver	2.6±0.8	2.9±1.1	2.5±0.8	2.7±0.5	0.663
MRglu heart	10.2±4.8	4.4±1.9***	8.9±4.0	7.2±2.1	0.003
MRglu abdominal adipose tissue	0.70±0.18	0.60±0.13*	0.58±0.22	0.50±0.11	0.204
MRglu leg muscles	5.7±3.3	6.9±3.1*	4.5±2.9	4.3±2.1	0.037

Data are presented as mean ± SD if normally distributed, otherwise geometric mean and 95% CI

^a^*p* values for change from baseline in surgery vs LCD group, ANCOVA test

^b^Glucose turnover data are from ^18^F-FDG-PET image assessment during clamp steady state

^*^p<0.05, ** *p*<0.01, *** *p*<0.001 for change from baseline within the group, paired *t* test

### Whole-body and tissue-specific glucose turnover assessed by PET

**Fig. 6:**
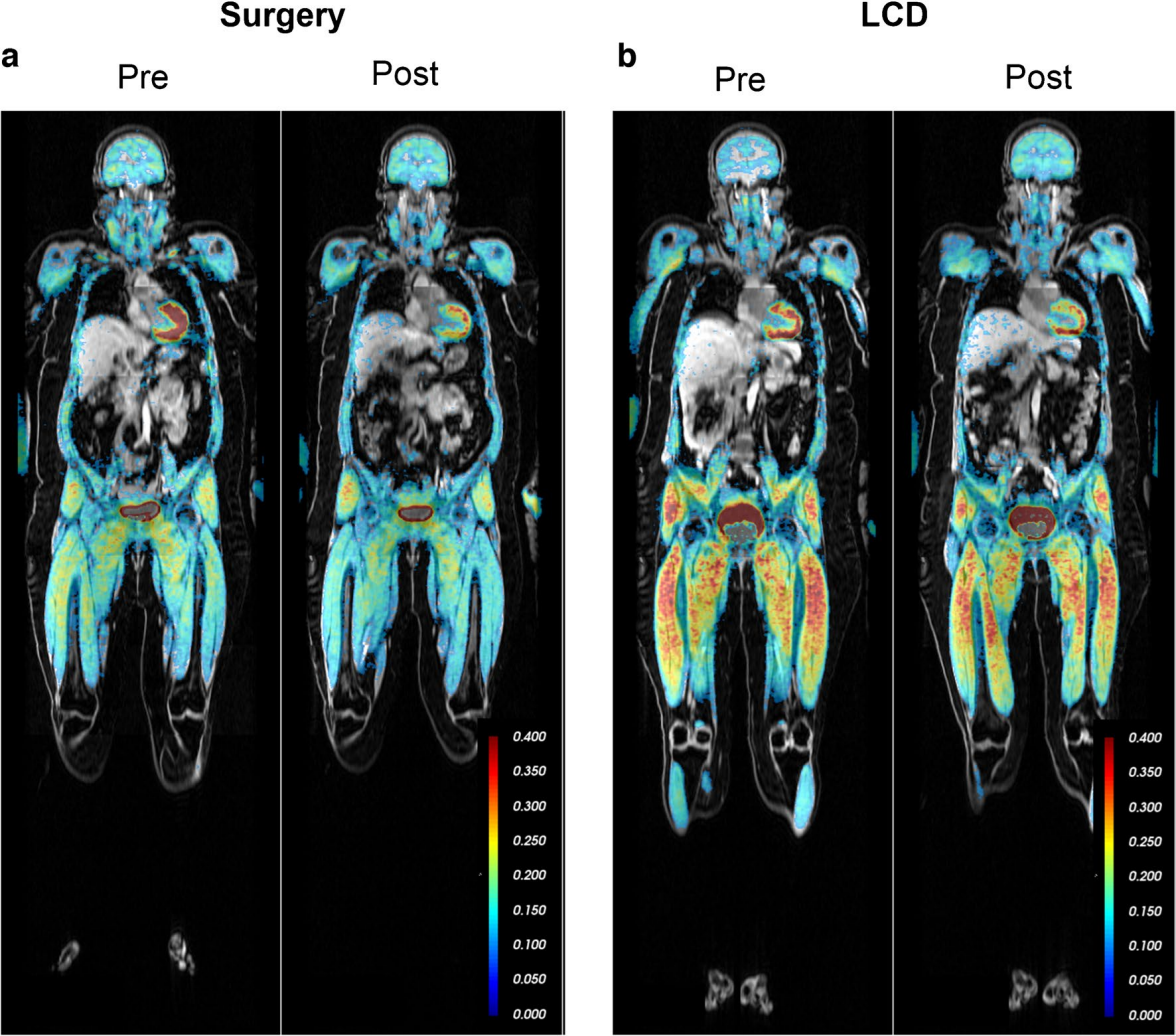
^18^F-FDG net influx rate Ki images depict whole-body glucose uptake rate (MRglu) during clamp steady state. Examples from the surgery group (**a**) and from the LCD group (**b**) are shown before (Pre) and after (Post) the respective intervention. The colour-bar indicates Ki values. Tissue-specific glucose uptake is quantified for brain, liver, heart, abdominal adipose tissue and leg muscles and are shown in Table 2

There was a non-significant reduction in Rd during steady-state hyperinsulinaemic–euglycaemic clamp following LCD (*p*=0.09). EGP remained unaltered after both interventions (Table 2). Following OS, there was a decrease in tissue-specific glucose uptake rate in the heart (MRglu heart, *p*<0.001) and abdominal adipose tissue (*p*<0.05), whereas there was an increase in the skeletal muscles of the legs (*p*<0.05). Neither of these effects was seen after LCD, and neither of the interventions affected glucose uptake rates in any of the other assessed tissues (i.e. brain or liver) (Table 2, Fig. 6). However, LCD led to a non-significant increase in the glucose uptake rate of the brain (Table 2).

### Correlation and sensitivity analyses

Reduction of fasting plasma glucose after intervention was positively associated with absolute and relative body weight loss following OS but not LCD (ESM Table 1). Furthermore, lowering of EGP was associated with body fat reduction, although this was significant only in the LCD group. The body fat % reduction negatively correlated with the liver fat reduction in the OS group, while in the LCD group, this association was positive (both *p*<0.05). No correlations were found between weight loss and glycaemic or ^18^F-FDG-PET/MRI-derived effect measures.

The two types of OS, RYGB and SG, lead to weight loss by partly differing mechanisms and they also display different effects on long-term diabetes remission and weight maintenance. Therefore, we performed separate sensitivity analyses by including only the larger subgroup undergoing RYGB. The short-term effects on glycaemia, insulin sensitivity, whole-body and tissue-specific fat content, and glucose turnover remained very similar, as did the limited differences when compared with LCD (data not shown).

## Discussion

This is the first study that directly compares the short-term effects of OS and LCD on tissue-specific glucose turnover and body composition. The early effects are of specific interest since OS rapidly improves glucose homeostasis (within a few days) in individuals with or without prior diabetes [11, 32], while LCDs can achieve similar effects but over several months [33]. We addressed body composition, glucose control and insulin action at both whole-body and tissue level in individuals with obesity and no diabetes. Integrated PET/MRI investigations during hyperinsulinaemic–euglycaemic clamps were conducted before and after the interventions. The two interventions exhibited partly similar short-term metabolic effects. However, despite nearly identical weight loss, there were some interesting differences: increased glucose uptake in skeletal muscle but reduced uptake in the heart and abdominal adipose tissue occurred after OS but not after an LCD.

### Clinical effects

OS and LCD induced a similar reduction in body weight (8–9 kg) 4 weeks after the intervention. Both groups of participants experienced comparable reductions in circulating lipids and BP, whereas fasting plasma glucose, insulin, HbA_1c_ and HOMA-IR were reduced more markedly, or only, following OS. After OS, a higher proportion of participants with prediabetes (impaired glucose tolerance or impaired fasting glucose) reverted to normoglycaemia, with four out of 10 (40%) becoming normoglycaemic compared with one out of seven (14%) for LCD. Taken together, the data support the notion that dysglycaemia normalises more rapidly after OS than after an LCD, and that this difference is independent of weight loss [11, 32].

### Body composition

OS and LCDs similarly reduced whole-body adipose tissue volume by about 7%, along with a reduction in liver fat content by 2% units, whereas pancreas fat did not change in either group. Interestingly, the participants that underwent OS exhibited a slightly greater volume reduction in non-adipose tissues than those given the LCD, suggesting a relatively greater loss of fluid from non-adipose vs adipose tissue in the OS compared with the LCD group. This has previously been reported as a rapid and possibly temporary effect following OS, although the mechanisms are not clear [34, 35].

Liver fat content was reduced similarly following both interventions; in the LCD group, this was positively correlated with the reduction in whole-body fat %. In contrast, the opposite was found with OS (i.e. a significant inverse relationship between whole-body and liver fat reduction). This might be explained by enhanced lipid release from adipose tissue, potentially leading to elevated uptake in the liver [36], which could in turn drive hepatic insulin resistance. Nonetheless, there was a net decrease in liver fat following OS, in keeping with lower HOMA-IR and fasting plasma glucose. Furthermore, the rapid decrease in fasting glucose and hepatic insulin resistance following OS is probably also due to several other factors, including resetting of secretion of incretins and other glucose-regulating hormones as well as adaptative changes of the brain’s glucose-sensing and regulating functions [16, 17, 29].

### Glucose turnover

In the OS group, there was a 20% increase in glucose AUC in response to an OGTT, in contrast to the LCD group. This result is likely attributed to the anatomical rearrangements specifically in RYGB patients, in whom the duodenum and much of the stomach are bypassed, allowing for rapid glucose absorption in the jejunum. During hyperinsulinaemia, only the OS group showed a significantly increased PET-derived glucose uptake in skeletal muscle (by about 25%). OS also led to a substantial 40% reduction in glucose uptake in the heart and a 10% reduction in abdominal adipose tissue, with no significant changes in the brain or liver.

Surprisingly, Rd and EGP were unaffected by either intervention. Previous work has shown reduced EGP post-RYGB, often under fasting conditions or at later time points than four weeks [37, 38]. Our data suggest a probable reduction in fasting EGP following RYGB, as evidenced by decreased fasting glucose and HOMA-IR. This implies that the glucose-lowering effect of OS is more related to fasting conditions than the fed state. At 4 weeks after OS, no clear improvements in glucose metabolism, insulin secretion or insulin action upon an oral glucose load were observed, as Matsuda and insulinogenic indices remained unchanged. This aligns with unaltered PET-derived glucose uptake at the whole-body level during hyperinsulinaemia. Nevertheless, the increased glucose uptake rate in skeletal muscle and adipose tissue supports the occurrence of rapid tissue-specific insulin sensitisation after OS [39].

### Integrated assessment

The outcomes of insulin sensitivity assessments displayed variability across diverse methods. Notably, the clamp experiments, coupled with PET/MRI investigations, reflected a submaximal insulin-stimulated state, a characteristic largely shared with the Matsuda index derived from OGTT data. Examining the impact of OS on insulin-stimulated glucose uptake in tissues, using FDG-PET, revealed distinctive patterns: an increased uptake in skeletal muscle and adipose tissue, and a reduced uptake in the heart. However, there were no significant changes in the whole-body glucose turnover, and those effects were also found following an LCD. In contrast, HOMA-IR reflects the fasting condition and suggests a more marked improvement of insulin resistance after OS.

Increased insulin sensitivity of skeletal muscle probably explains the observed increase in its glucose utilisation at 4 weeks after OS, as previously observed in other studies [40]. However, our data also revealed that glucose uptake was reduced in adipose tissue and myocardium. The latter might be due to lower cardiac output [41] and hence less strain on the heart. We hypothesise that the rapid glycaemic improvement after OS is mainly accounted for by reduced hepatic glucose production in the fasting state. However, this hypothesis was not directly addressed as EGP was only measured during hyperinsulinaemic clamps and did not change. Albeit direct measurements were not performed, the notion of lowered EGP during fasting [38], and thus much of everyday life, is supported by the observed reduction in HOMA-IR after OS. Notably, at later time points after OS, there are clear effects also on whole-body glucose utilisation during hyperinsulinaemia [17, 42].

Our correlation analyses indicate that the magnitude of body weight loss does not directly impact the rapid changes in glycaemic regulation. This is also supported by the lowering of glycaemic measures seen 4 weeks after OS but not LCD, despite similar body weight and fat loss. Thus, we propose that, after OS compared with LCD, there is a more rapid change of fasting plasma glucose and this may be mediated by a lower hepatic glucose production in the fasting state, which could not be assessed during our hyperinsulinaemic clamps.

We observed no evident effects on whole-body insulin action during the clamps, as reflected by the *M* value (glucose infusion rate) and glucose uptake rates derived from FDG-PET. This finding was unexpected as both weight loss per se and, in particular, OS are known to markedly reverse insulin resistance. However, it seems likely that those effects require several months to manifest. This was previously shown by us and others and may involve adipose tissue remodelling and accompanying functional changes including insulin responsiveness and altered gene expression [12, 17]. Moreover, the brain and neuroendocrine pathways may play an important role. Attenuated insulin-antagonistic neurohormonal responses (adrenocorticotropic hormone, cortisol, glucagon, growth hormone and sympathoadrenergic) have been shown by us and others following RYGB [16, 43]. Within the brain, glucose utilisation, as well as blood flow and neural network activity, is changed and these alterations likely contribute to the resetting of whole-body glucose metabolism. Such effects (e.g. a general reduction in brain glucose uptake) were demonstrated after at least 4 months and thus may not operate earlier [16, 29, 44]. This is supported by our present finding of an unchanged rate of glucose uptake in the whole brain after both OS and LCD. However, this does not preclude some changes in critical brain regions or effects on other brain functions.

### Strengths and limitations

This study is the first to compare the short-term metabolic effects of OS and LCD with a comprehensive assessment of tissue-specific and whole-body glucose turnover. Integrated simultaneous PET/MRI imaging was employed for the measurement of body composition, fat distribution and quantitative assessment of glucose fluxes. Notably, PET/MRI offers advantages over the more commonly available and less costly PET/computed tomography (CT) method, thanks to its lower radiation dose for whole-body investigations. Furthermore, MRI enables more precise quantifications such as liver fat compared with CT [45]. While our study provides valuable insights, we acknowledge inherent limitations due to the modest sample size, which was due to challenges in participant recruitment and resource constraints, in turn posed by the complex and cumbersome investigations. Consequently, there is low statistical power to detect minor differences in effects between the two interventions. Additionally, since most of the participants in the study were female, generalisations to all sex and genders in the populations is limited.

Of note, we only address the first 4 weeks after interventions. Repeated follow-up investigations would be of interest but were hindered by the waiting list design (with surgery performed directly after the study in the LCD group) and also by radiation exposure limits. At this point, we have not analysed incretin hormones, which may be involved in the early effects on glucose homeostasis, particularly following OS where enhanced responses to oral glucose are found [12, 44]. However, stimulated insulin secretion during OGTT was not amplified following either treatment, so the role of incretins in short-term glycaemic changes may be limited. In future work, we will address gut hormone regulation after LCD and RYGB. Quantitative glucose partitioning to different tissues should also be explored in more detail but would require rather challenging volume determination of each tissue of interest [26].

### Conclusions

While achieving identical weight loss, OS and LCD also led to similar reductions in whole-body and liver fat after 4 weeks. However, only surgery increased the glucose uptake rate in skeletal muscle during hyperinsulinaemia, whereas it reduced the uptake rate in the myocardium and abdominal adipose tissue. Both liver and brain glucose uptake rates were similar between OS and LCD, as were whole-body glucose uptake and EGP. Taken together, the data indicate that the short-term metabolic effects of OS are not explained by loss of body fat alone. Although the full effects on whole-body insulin resistance and blood glucose levels do not occur immediately, there are clear reductions in fasting plasma glucose and insulin resistance as well as in HbA_1c_ as early as 4 weeks post-OS, potentially contributing to the prevention or reversal of type 2 diabetes. This indicates a specific and rapid glucose-lowering effect of OS, probably mainly explained by lower glucose production by the liver in the non-fed condition.

### Supplementary Information

Below is the link to the electronic supplementary material.

ESM (PDF 102 KB)

## Data Availability

The datasets generated during and/or analysed during the current study are available from the corresponding authors upon reasonable request.

## Abbreviations

AST: Aspartate aminotransferase
CONSORT: Consolidated Standards of Reporting Trials
CT: Computed tomography
EGP: Endogenous glucose production
^18^F-FDG: [^18^F]fluorodeoxyglucose
IDEAL-IQ: Iterative decomposition of fat and water with echo asymmetry and least squares estimation
LBM: Lean body mass
LCD: Low-energy (low-calorie) diet
MRAC: Magnetic resonance attenuation correction
MRglu: Tissue glucose metabolic rate
OS: Obesity surgery
PET: Positron emission tomography
Rd: Glucose disposal
RYGB: Roux-en-Y gastric bypass
SAT: Subcutaneous adipose tissue
SG: Sleeve gastrectomy
