# Supplementary material for: Short-term effects of obesity surgery versus low-energy diet on body composition and tissue-specific glucose uptake: a randomised clinical study using whole-body integrated 18F-FDG-PET/MRI
Source: Diabetologia. 2024 Apr 24;67(7):1399–412. doi: 10.1007/s00125-024-06150-3 (PMC11153296; doi:10.1007/s00125-024-06150-3)
Supplement: Supplementary file 1 — ESM (PDF 102 KB) [file 125_2024_6150_MOESM1_ESM.pdf]

# ESM Methods

## Experimental design

The weight loss during the first postoperative month after gastric bypass (GBP) surgery is known to be very similar to that obtained after 4 weeks of treatment with a low-energy diet (low-calorie diet, LCD), and both interventions were expected to result in approximately 8-10% weight loss [1, 2].

By clinical routines, the LCD group underwent obesity surgery directly after the diet intervention and study completion. During surgery, subcutaneous adipose tissue (SAT), omental adipose tissue (OAT), and abdominal muscle biopsies were also collected, and results from these will be reported separately. Blood samples were analysed for plasma glucose and lipids, and serum insulin and C-peptide, using established routine methods at the Clinical Chemistry Department at Uppsala University Hospital. During 4-day periods before and after the interventions, participants wore accelerometers (Philips Respironics Actical, Philips Respironics, Murrysville, PA, USA) on their right ankle to assess changes in 24-hour physical activity and energy expenditure.

## PET/MRI-derived quantifications

### *Quantification of $^{18}$F-FDG net glucose influx rate and glucose turnover from PET*

The quantitative $^{18}$F-FDG net influx rate $K_i$ images were created by using the irreversible two-tissue compartment Patlak method for each voxel. An image-derived whole-blood time-activity curve was generated from the 10-minute dynamic PET scan over the thorax by applying a volume of interest in the ascending aorta and extended to the full length of the PET protocol by 7 discrete arterialized blood samples. The image-derived input function was corrected for binding to plasma proteins and platelets by using the mean ratio of blood and plasma radioactivity concentration in the arterialized blood samples. The voxel-wise Patlak analysis was performed in Matlab (Matlab 2018b). Glucose turnover at the whole-body level (whole-body glucose disposal, Rd; and endogenous glucose production, EGP) and in specific tissues (Mrglu) was calculated based on $^{18}$F-FDG kinetics, tissue lumped constants, and adjustment for urinary and blood $^{18}$F-FDG, as previously reported [3, 4].

### *Quantification of adipose and non-adipose tissue volumes from whole-body water-fat MRI*

Adipose and non-adipose tissue volumes were quantified from whole-body water-fat MRI using an automated image analysis approach. The analysis first combined the water and fat signal images into a sum image, in which the body was segmented using intensity thresholding. This method utilized a 3-class Otsu-based technique, which roughly separated the sum image into the background, non-adipose tissue, and adipose tissue. The inverse of the background segmentation was defined as body segmentation. A water bottle, occasionally positioned close to the calves and feet to improve the originally low signal-to-noise ratio of these structures, needed to be removed from the body segmentations. This was achieved by morphological operations where only the largest connected component, i.e. the body, was kept. Quantitative fat fraction (FF) images were calculated by dividing the fat signal image by the sum image within the body segmentation. Thereafter, the body segmentation was separated into adipose tissue and non-adipose tissue, by thresholding of FF at 50%. The total volumes were calculated as the number of voxels in each class multiplied by the voxel volume.

### *Quantification of liver and pancreatic fat % from MRI*

The FF images from the dedicated water-fat MRI acquisitions of the liver and pancreas were used for the assessment of organ-specific fat %. Delineation of the two organs was performed manually, for each slice, on the FF maps. The two software programs ImageJ (version 1.52) [5] and 3DSlicer (version 4.11) [6] were used for the delineation of the liver and pancreas, respectively. For each subject, the images of baseline and post-intervention were delineated side-by-side for increased standardization of the segmentations between visits. Care was taken to exclude border voxels, to reduce partial volume effects in the FF measurement. In addition, the three outermost slices in the head and feet direction were omitted to avoid the influence of suboptimal slice profiles in the measurement. The median FF within the multi-slice segmentation was used as a measure of fat %.

## References

[1] Campos GM, Rabl C, Peeva S, et al. (2010) Improvement in Peripheral Glucose Uptake After Gastric Bypass Surgery Is Observed Only After Substantial Weight Loss Has Occurred and Correlates with the Magnitude of Weight Lost. Journal of Gastrointestinal Surgery 14(1): 15-23. 10.1007/s11605-009-1060-y

[2] Salem V, Demetriou L, Behary P, et al. (2021) Weight Loss by Low-Calorie Diet Versus Gastric Bypass Surgery in People With Diabetes Results in Divergent Brain Activation Patterns: A Functional MRI Study. Diabetes Care 44(8): 1842-1851. 10.2337/dc20-2641
[3] Eriksson JW, Visvanathar R, Kullberg J, et al. (2021) Tissue-specific glucose partitioning and fat content in prediabetes and type 2 diabetes: whole-body PET/MRI during hyperinsulinemia. European Journal of Endocrinology 184(6): 879-889. 10.1530/EJE-20-1359
[4] Rebelos E, Immonen H, Bucci M, et al. (2019) Brain glucose uptake is associated with endogenous glucose production in obese patients before and after bariatric surgery and predicts metabolic outcome at follow-up. Diabetes, Obesity and Metabolism 21(2): 218-226. 10.1111/dom.13501
[5] Schneider CA, Rasband WS, Eliceiri KW (2012) NIH Image to ImageJ: 25 years of Image Analysis. Nature methods 9(7): 671-675
[6] Fedorov A, Beichel R, Kalpathy-Cramer J, et al. (2012) 3D Slicer as an Image Computing Platform for the Quantitative Imaging Network. Magnetic resonance imaging 30(9): 1323-1341. 10.1016/j.mri.2012.05.001

**ESM Table 1 –** Correlations between change from baseline (delta) after obesity surgery and low-energy diet (low-calorie diet, LCD) in adiposity measures vs change in glycaemia, glucose turnover, and body composition.

| | Delta BMI | | Delta body weight % | | Delta body fat % | | Delta Waist/hip ratio | |
|---|---|---|---|---|---|---|---|---|
| | Rho | *p* value | Rho | *p* value | Rho | *p* value | Rho | *p* value |
| **Obesity surgery** **Change from baseline (delta)** | | | | | | | | |
| ***Glucose metabolism*** | | | | | | | | |
| HbA1c | 0.283 | 0.308 | 0.209 | 0.454 | -0.344 | 0.209 | -0.195 | 0.487 |
| Fasting P-glucose | **0.728** | **0.002** | **0.621** | **0.013** | -0.170 | 0.545 | -0.171 | 0.543 |
| HOMA-IR | -0.036 | 0.899 | 0.093 | 0.742 | -0.222 | 0.427 | -0.211 | 0.451 |
| Fasting S-insulin | -0.036 | 0.899 | 0.093 | 0.742 | -0.222 | 0.427 | -0.211 | 0.451 |
| 2-h post-OGTT glucose | -0.075 | 0.799 | 0.099 | 0.737 | *0.506* | *0.065* | -0.042 | 0.887 |
| Insulinogenic index | 0.141 | 0.616 | 0.207 | 0.459 | -0.152 | 0.589 | -0.318 | 0.248 |
| Disposition index | -0.070 | 0.805 | -0.111 | 0.694 | 0.139 | 0.620 | 0.154 | 0.585 |
| Matsuda index | 0.041 | 0.884 | -0.061 | 0.830 | 0.193 | 0.491 | *0.450* | *0.092* |
| M-value | -0.245 | 0.379 | -0.282 | 0.308 | 0.433 | 0.107 | 0.004 | 0.990 |
| ***Whole-body glucose turnover*** | | | | | | | | |
| GIR | -0.225 | 0.420 | -0.254 | 0.362 | 0.304 | 0.271 | 0.021 | 0.940 |
| EGP | 0.281 | 0.311 | 0.161 | 0.567 | -0.440 | 0.101 | 0.300 | 0.277 |
| Rd | -0.068 | 0.810 | -0.093 | 0.742 | 0.141 | 0.616 | 0.375 | 0.168 |
| ***Tissue glucose uptake*** | | | | | | | | |
| MRglu brain | -0.041 | 0.884 | -0.082 | 0.771 | -0.252 | 0.365 | -0.411 | 0.128 |
| MRglu liver | -0.338 | 0.218 | *-0.450* | *0.092* | -0.241 | 0.386 | -0.261 | 0.348 |
| MRglu heart | 0.236 | 0.397 | 0.225 | 0.420 | -0.179 | 0.524 | 0.068 | 0.810 |
| MRglu abdominal adipose tissue | -0.088 | 0.756 | -0.168 | 0.550 | *-0.447* | *0.095* | 0.125 | 0.657 |
| MRglu leg muscles | -0.114 | 0.685 | -0.271 | 0.328 | 0.052 | 0.854 | 0.314 | 0.254 |
| ***MR-derived body volumes*** | | | | | | | | |
| Whole body volume | **0.670** | **0.006** | **0.825** | **<.001** | 0.170 | 0.545 | -0.132 | 0.639 |
| Adipose tissue volume | 0.204 | 0.466 | 0.307 | 0.265 | **0.586** | **0.022** | -0.200 | 0.475 |
| Non-adipose tissue volume | **0.611** | **0.015** | **0.618** | **0.014** | **-0.550** | **0.033** | -0.107 | 0.704 |

| | | | | | | | | |
|---|---|---|---|---|---|---|---|---|
| ***Tissue Fat %*** | | | | | | | | |
| Liver | 0.304 | 0.271 | 0.254 | 0.362 | **-0.529** | **0.043** | -0.182 | 0.516 |
| Pancreas | -0.164 | 0.558 | -0.150 | 0.594 | 0.202 | 0.470 | **-0.607** | **0.016** |
| **LCD<br>Change from baseline (delta)** | | | | | | | | |
| ***Glucose metabolism*** | | | | | | | | |
| HbA1c | 0.111 | 0.777 | 0.238 | 0.537 | 0.221 | 0.567 | 0.375 | 0.321 |
| Fasting P-glucose | 0.042 | 0.915 | 0.025 | 0.949 | 0.209 | 0.589 | -0.259 | 0.500 |
| HOMA-IR | 0.217 | 0.576 | 0.250 | 0.516 | 0.183 | 0.637 | -0.050 | 0.898 |
| Fasting S-insulin | 0.367 | 0.332 | 0.383 | 0.308 | 0.217 | 0.576 | 0.000 | 1.000 |
| 2-h post-OGTT glucose | 0.000 | 1.000 | 0.126 | 0.748 | 0.678 | 0.045 | -0.109 | 0.781 |
| Insulinogenic index | 0.133 | 0.732 | 0.133 | 0.732 | 0.150 | 0.700 | -0.383 | 0.308 |
| Disposition index | -0.067 | 0.865 | -0.167 | 0.668 | -0.117 | 0.765 | 0.017 | 0.966 |
| Matsuda index | -0.367 | 0.332 | -0.367 | 0.332 | 0.133 | 0.732 | -0.117 | 0.765 |
| M-value | 0.071 | 0.867 | 0.000 | 1.000 | -0.476 | 0.233 | -0.452 | 0.260 |
| ***Whole-body glucose turnover*** | | | | | | | | |
| GIR | 0.095 | 0.823 | -0.024 | 0.955 | -0.548 | 0.160 | -0.405 | 0.320 |
| EGP | -0.357 | 0.385 | -0.381 | 0.352 | **0.905** | **0.002** | 0.048 | 0.911 |
| Rd | -0.381 | 0.352 | -0.429 | 0.289 | 0.071 | 0.867 | -0.476 | 0.233 |
| ***Tissue glucose uptake*** | | | | | | | | |
| MRglu brain | -0.095 | 0.823 | 0.024 | 0.955 | 0.524 | 0.183 | 0.452 | 0.260 |
| MRglu liver | -0.071 | 0.867 | -0.048 | 0.911 | -0.143 | 0.736 | **0.857** | **0.007** |
| MRglu heart | -0.071 | 0.867 | 0.143 | 0.736 | -0.095 | 0.823 | 0.571 | 0.139 |
| MRglu abdominal adipose tissue | -0.071 | 0.867 | -0.310 | 0.456 | 0.214 | 0.610 | 0.262 | 0.531 |
| MRglu leg muscles | -0.333 | 0.420 | -0.476 | 0.233 | -0.095 | 0.823 | -0.333 | 0.420 |
| ***MR-derived body volumes*** | | | | | | | | |
| Whole body volume | 0.333 | 0.420 | 0.262 | 0.531 | 0.19 | 0.651 | 0.095 | 0.823 |
| Adipose tissue volume | 0.286 | 0.493 | 0.167 | 0.693 | -0.024 | 0.955 | 0.119 | 0.779 |
| Non-adipose tissue volume | -0.095 | 0.823 | 0.024 | 0.955 | 0.500 | 0.207 | -0.190 | 0.651 |
| ***Tissue Fat %*** | | | | | | | | |
| Liver | -0.167 | 0.693 | -0.119 | 0.779 | **0.714** | **0.047** | 0.119 | 0.779 |

| | | | | | | | | |
|---|---|---|---|---|---|---|---|---|
| Pancreas | 0.310 | 0.456 | 0.262 | 0.531 | 0.190 | 0.651 | -0.167 | 0.693 |

OS, obesity surgery (n=14-15); LCD, low-calorie diet (n=8-9); BMI, body mass index; HbA1c, glycated haemoglobin; HOMA-IR, HOMA IR, homeostatic model assessment of insulin resistance index (fasting blood glucose * fasting insulin / 22.5); MR, magnetic resonance; GIR, glucose infusion rate; EGP, endogenous glucose production; Rd, whole-body glucose uptake; MRglu, tissue glucose metabolic rates; P-, plasma glucose; S-, serum.
